# Supplementary material for: Higher Periwound Temperature Associated with Wound Healing of Pressure Ulcers Detected by Infrared Thermography
Source: J Clin Med. 2021 Jun 29;10(13):2883. doi: 10.3390/jcm10132883 (PMC8269037; doi:10.3390/jcm10132883)

Figure S1: Case demostrations with temperature and wound condition

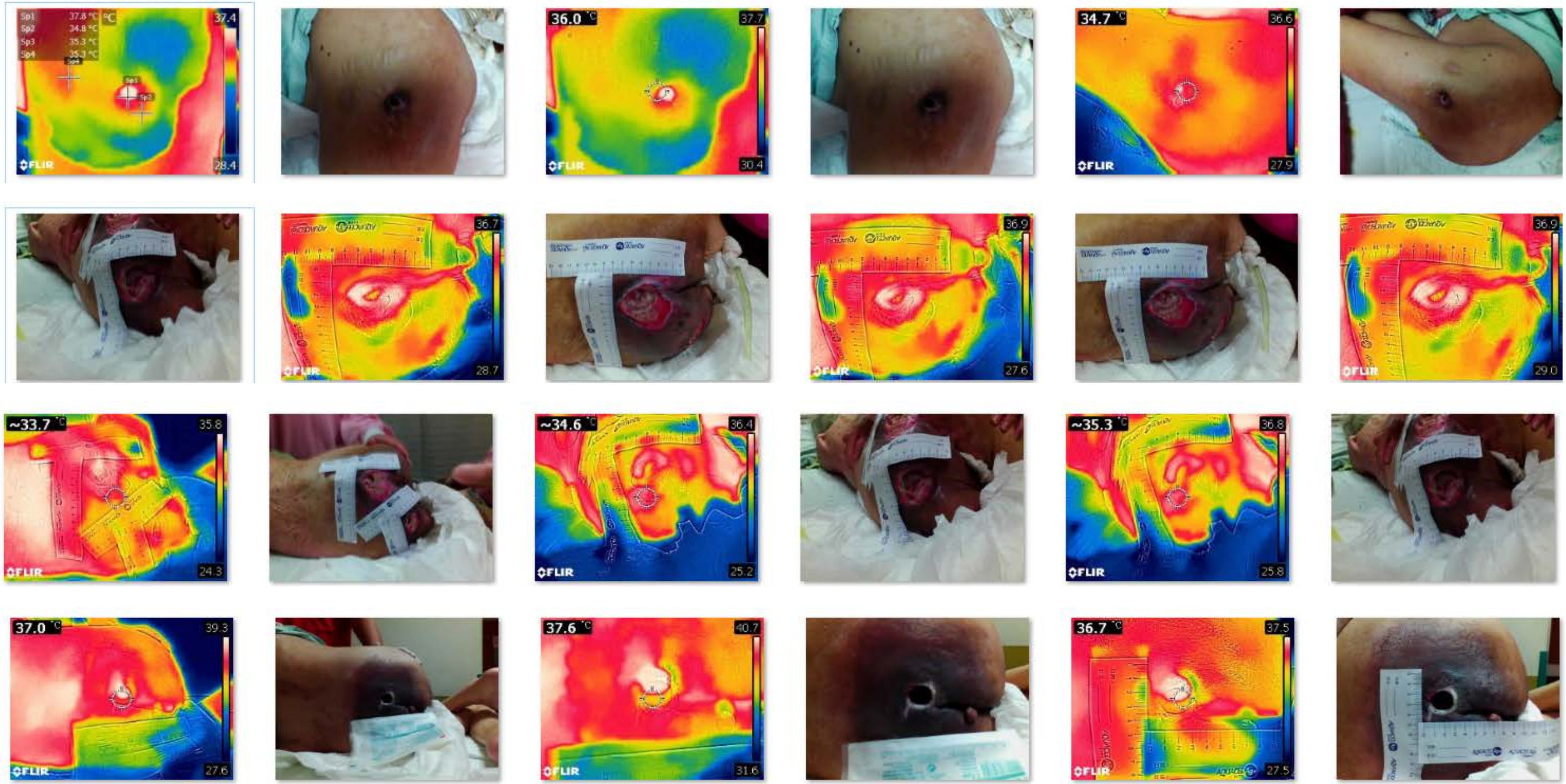

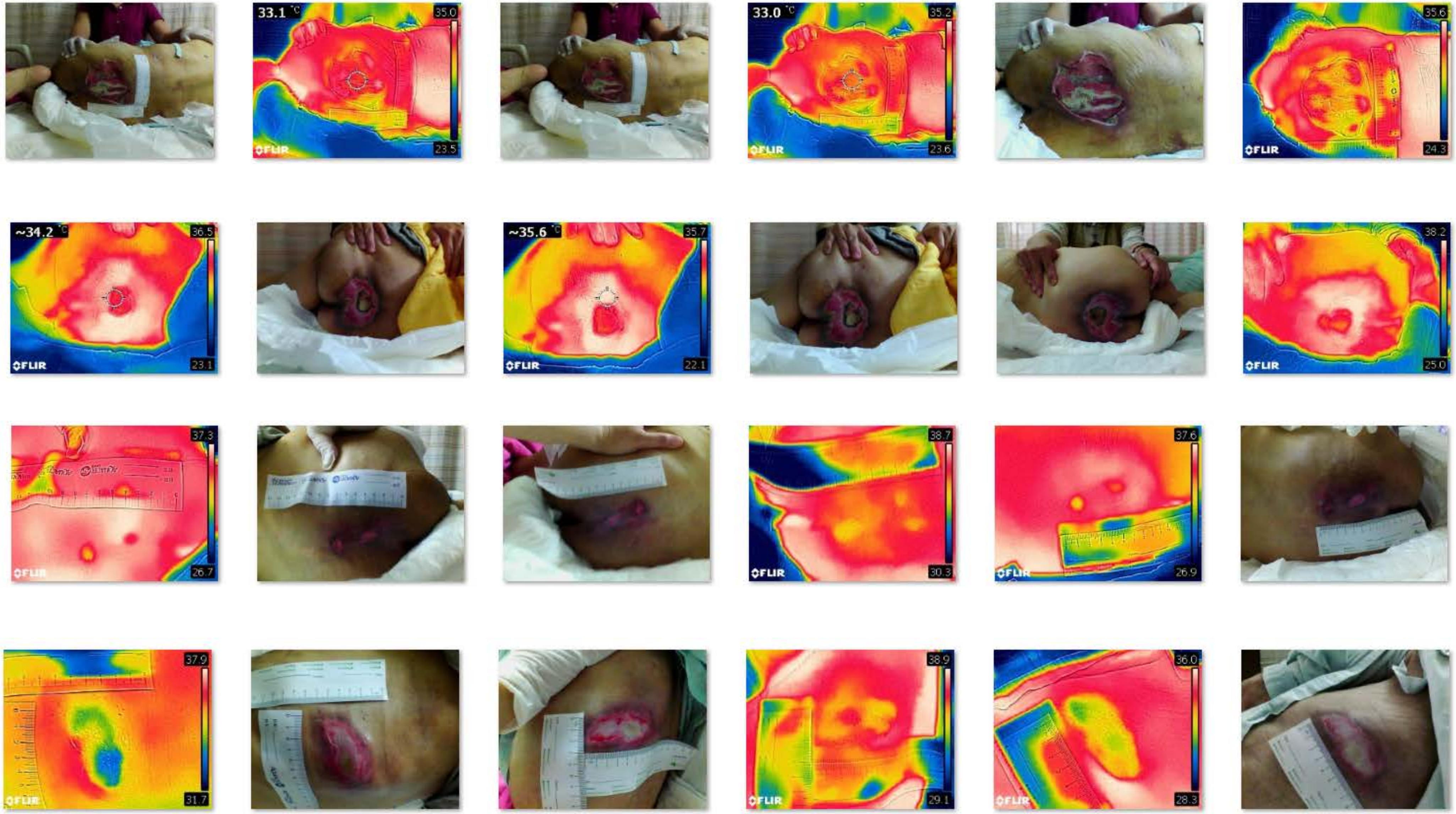
33.1 °C
35.0
23.5
FLIR
33.0 °C
35.2
23.6
FLIR
35.6
24.3
FLIR
~34.2 °C
36.5
23.1
FLIR
~35.6 °C
35.7
22.1
FLIR
38.2
25.0
FLIR
37.3
26.7
38.7
30.3
FLIR
37.6
26.9
37.9
31.7
38.9
29.1
FLIR
36.0
28.3

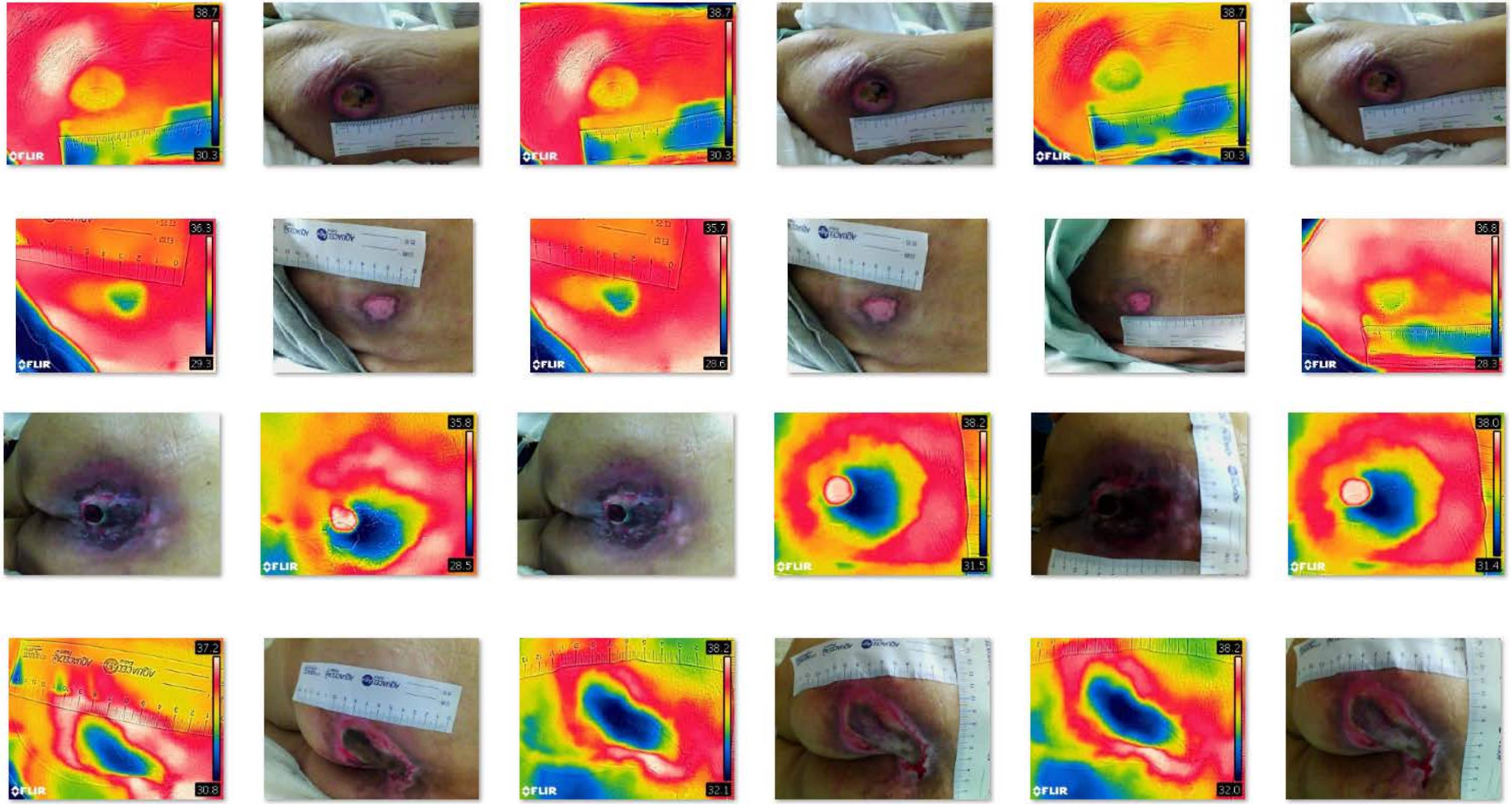
FLIR
38.7
30.3
38.7
30.3
38.7
30.3
36.3
29.3
35.7
28.6
36.8
28.3
35.8
28.5
38.2
31.5
38.0
31.4
37.2
30.8
38.2
32.1
38.2
32.0

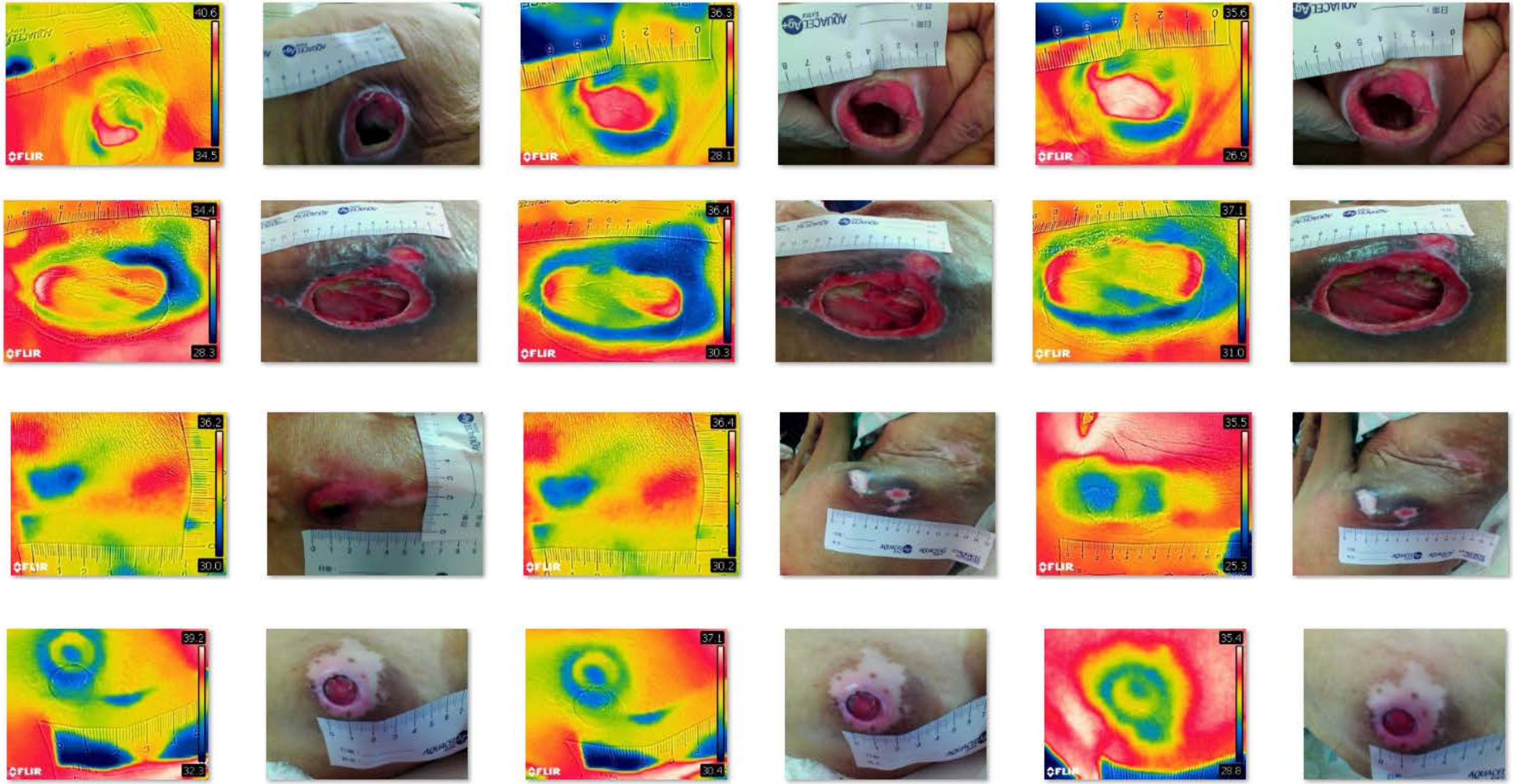
40.6
34.5
36.3
28.1
35.6
26.9
34.4
28.3
36.4
30.3
37.1
31.0
36.2
30.0
36.4
30.2
35.5
25.3
39.2
32.3
37.1
30.4
35.4
28.8

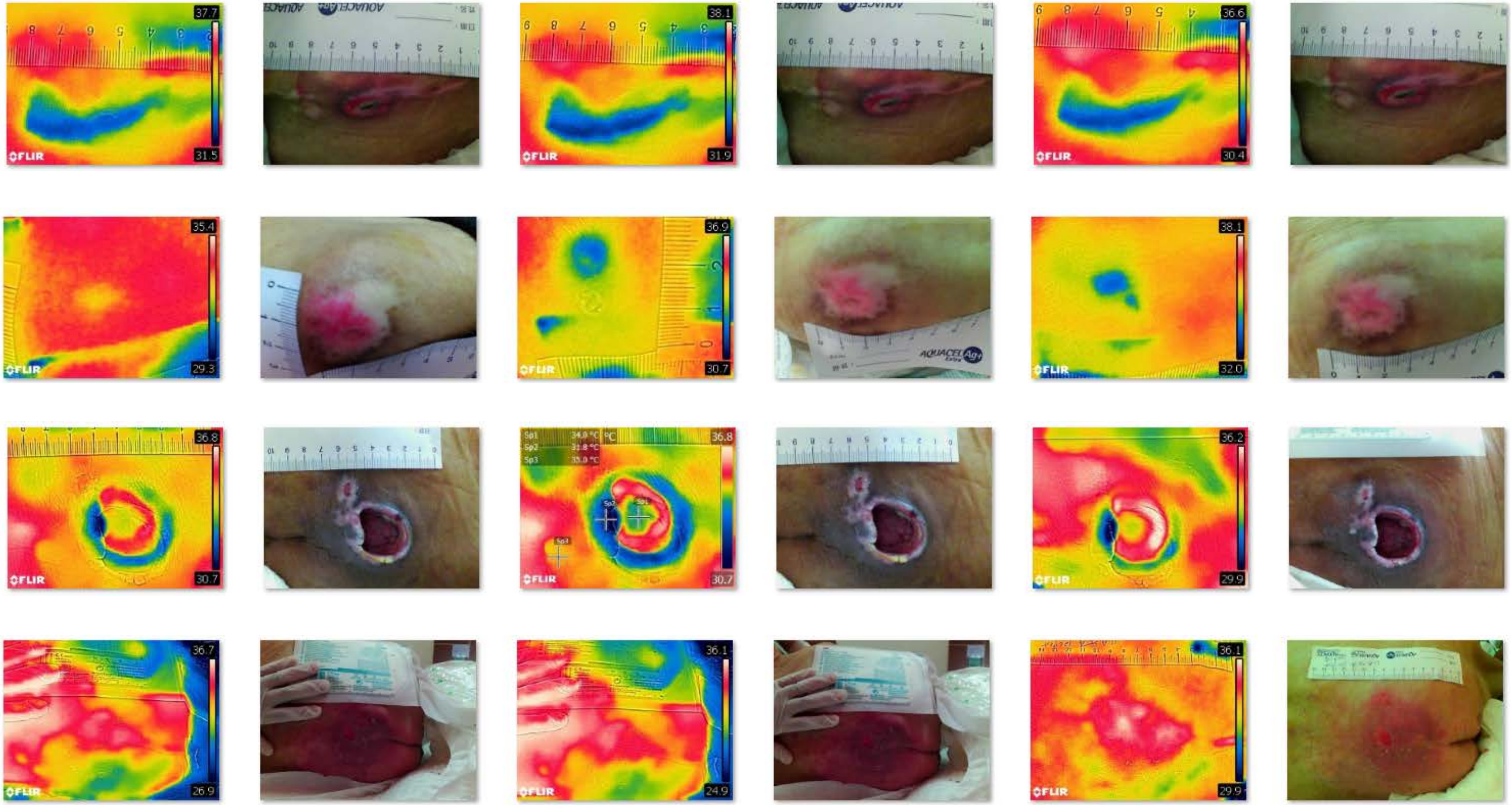
37.7
31.5
38.1
31.9
36.6
30.4
35.4
29.3
36.9
30.7
38.1
32.0
36.8
30.7
36.8
30.7
36.2
29.9
36.7
26.9
36.1
24.9
36.1
29.9

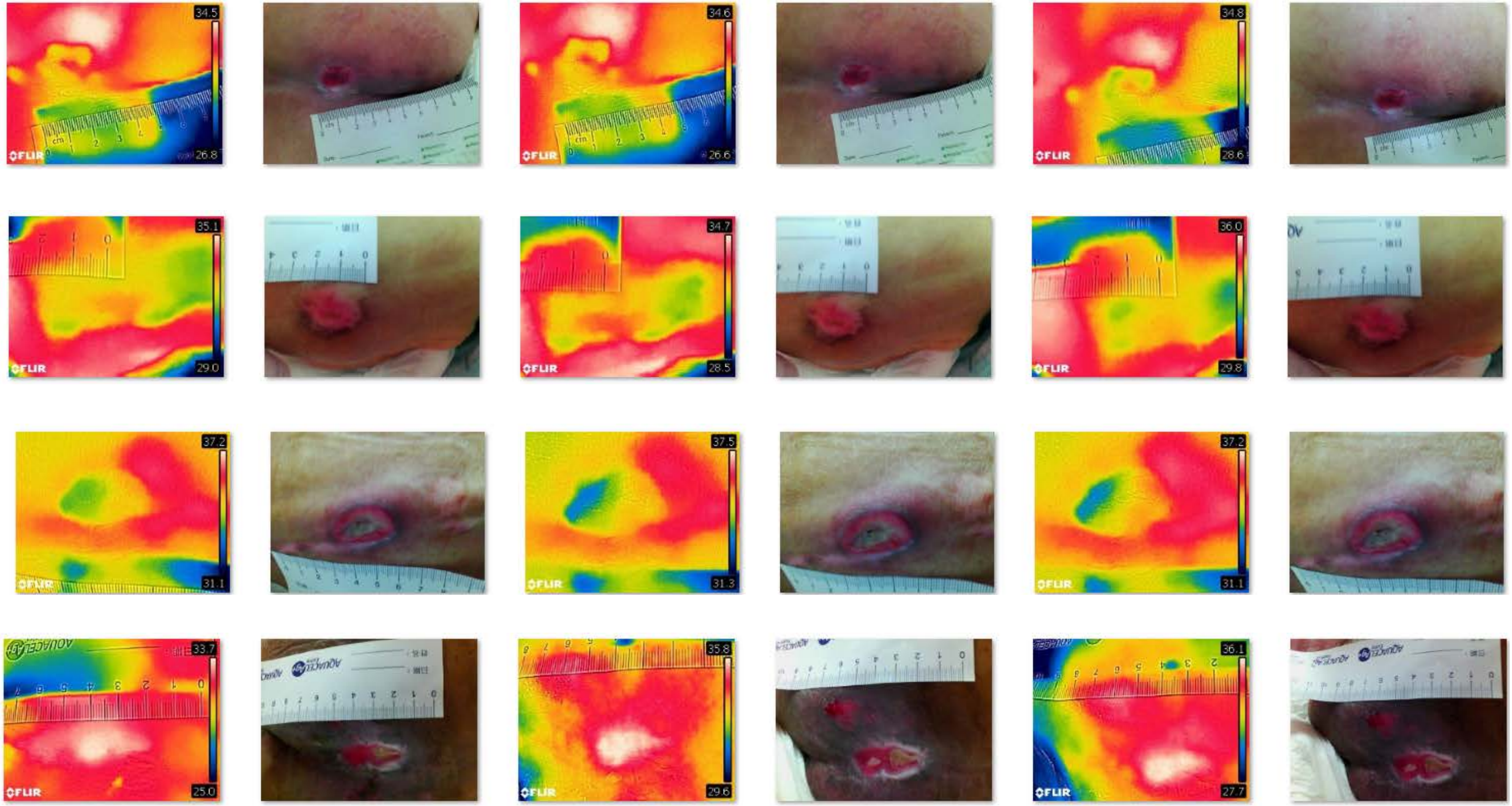
34.5
26.8
FLIR
34.6
26.6
FLIR
34.8
28.6
FLIR
35.1
29.0
FLIR
34.7
28.5
FLIR
36.0
29.8
FLIR
37.2
31.1
FLIR
37.5
31.3
FLIR
37.2
31.1
FLIR
33.7
25.0
FLIR
AQUACEL
35.8
29.6
FLIR
36.1
27.7
FLIR
AQUACEL

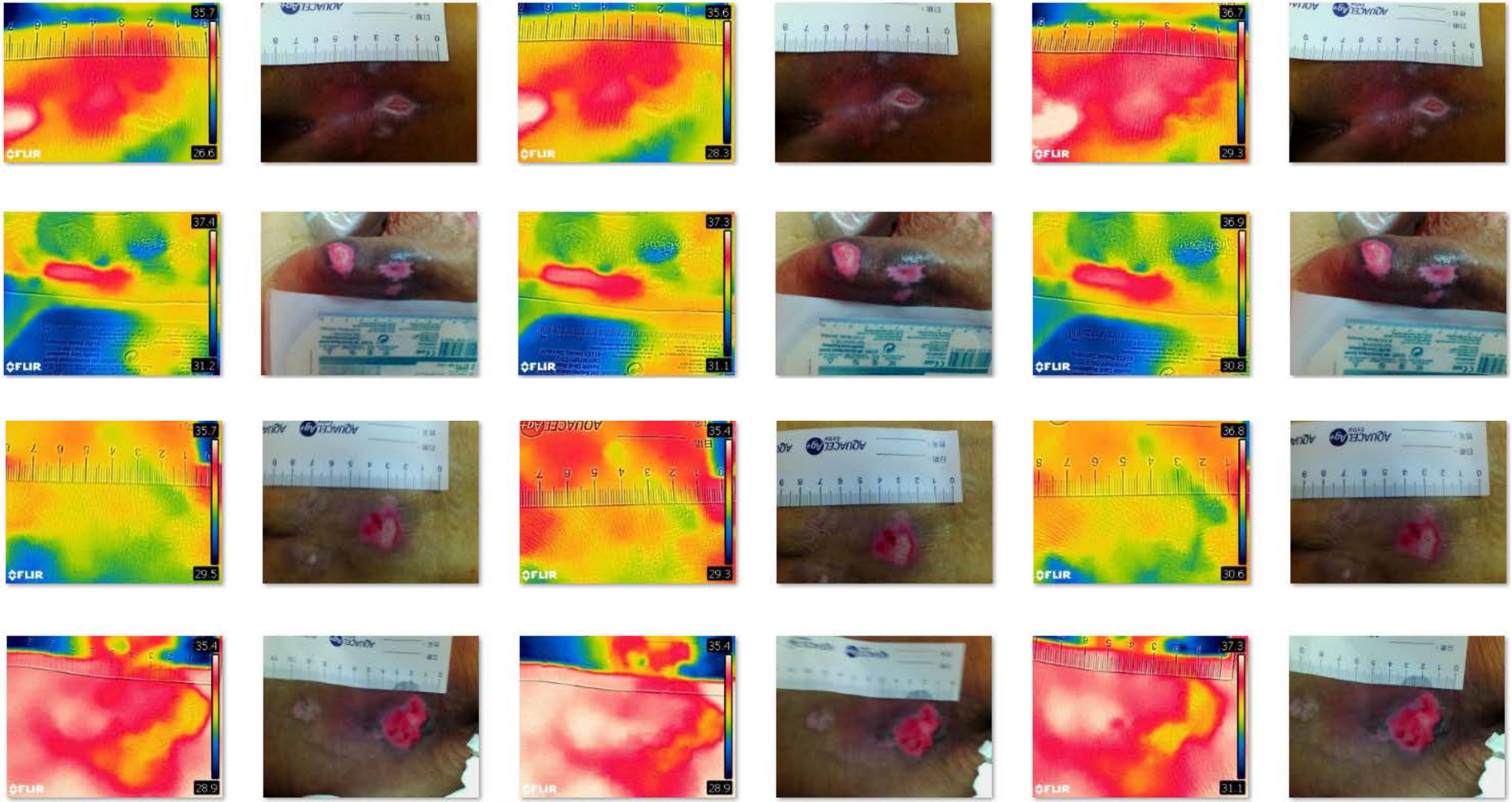
35.7
26.6
35.6
28.3
36.7
29.3
37.4
31.2
37.3
31.1
36.9
30.8
35.7
29.5
35.4
29.3
36.8
30.6
35.4
28.9
35.4
28.9
37.3
31.1
FLIR
AQUACEL Ag+

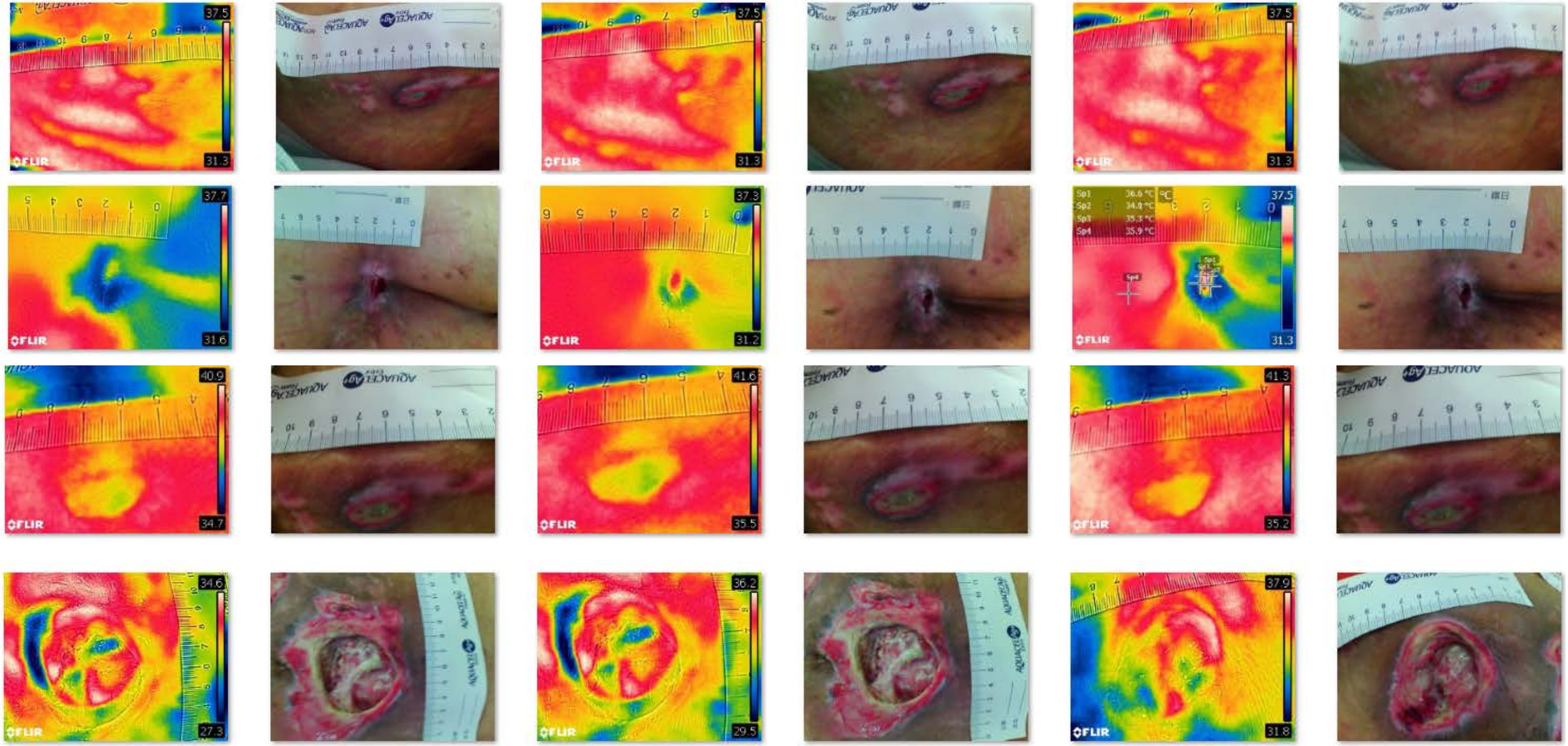
37.5
31.3
37.5
31.3
37.5
31.3
37.7
31.6
37.3
31.2
Sp1 36.6 °C
Sp2 34.8 °C
Sp3 35.3 °C
Sp4 35.9 °C
37.5
31.3
40.9
34.7
41.6
35.5
41.3
35.2
34.6
27.3
36.2
29.5
37.9
31.8

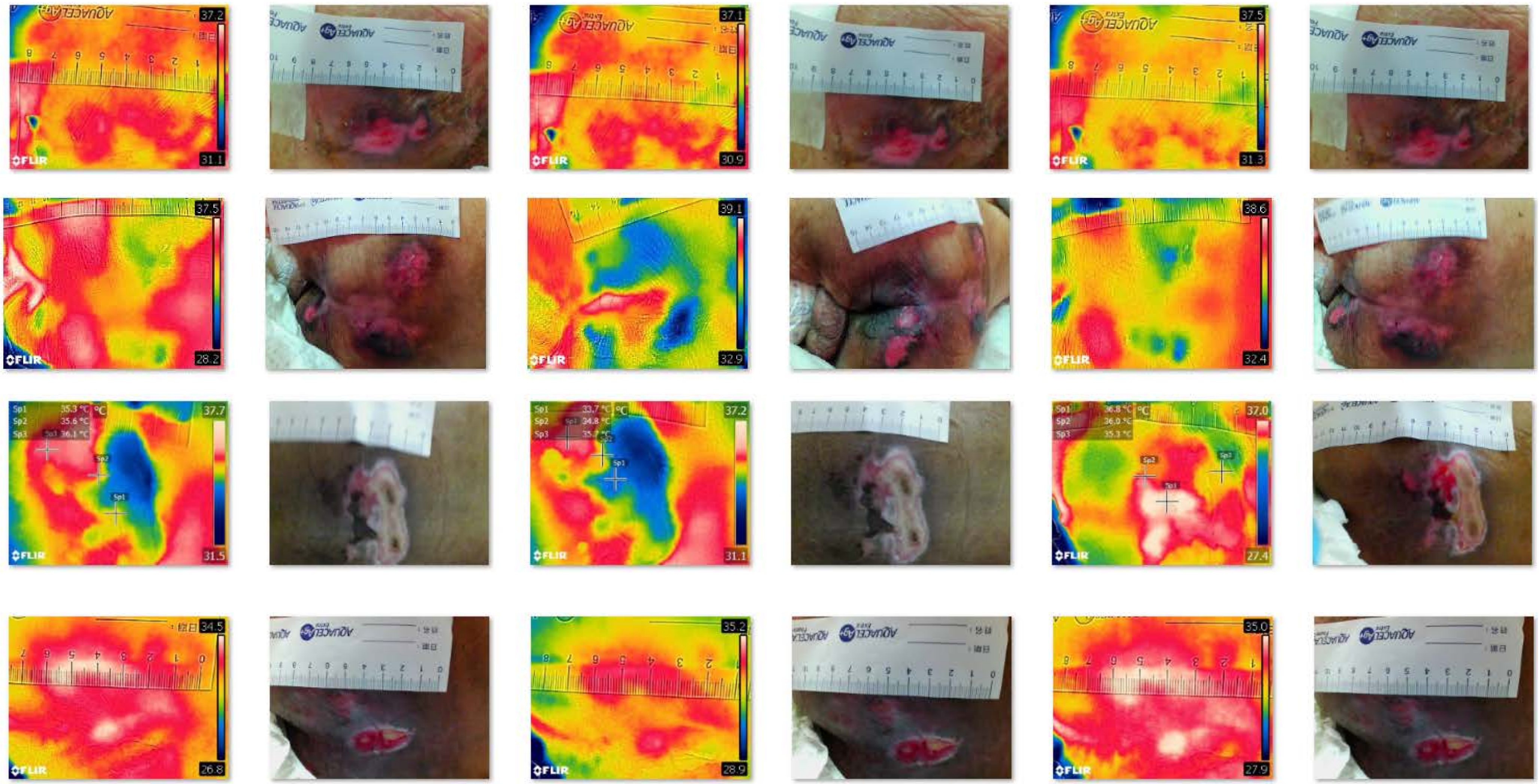
37.2
31.1
FLIR
37.1
30.9
37.5
31.3
37.5
28.2
39.1
32.9
38.6
32.4
37.7
31.5
37.2
31.1
37.0
27.4
34.5
26.8
35.2
28.9
35.0
27.9

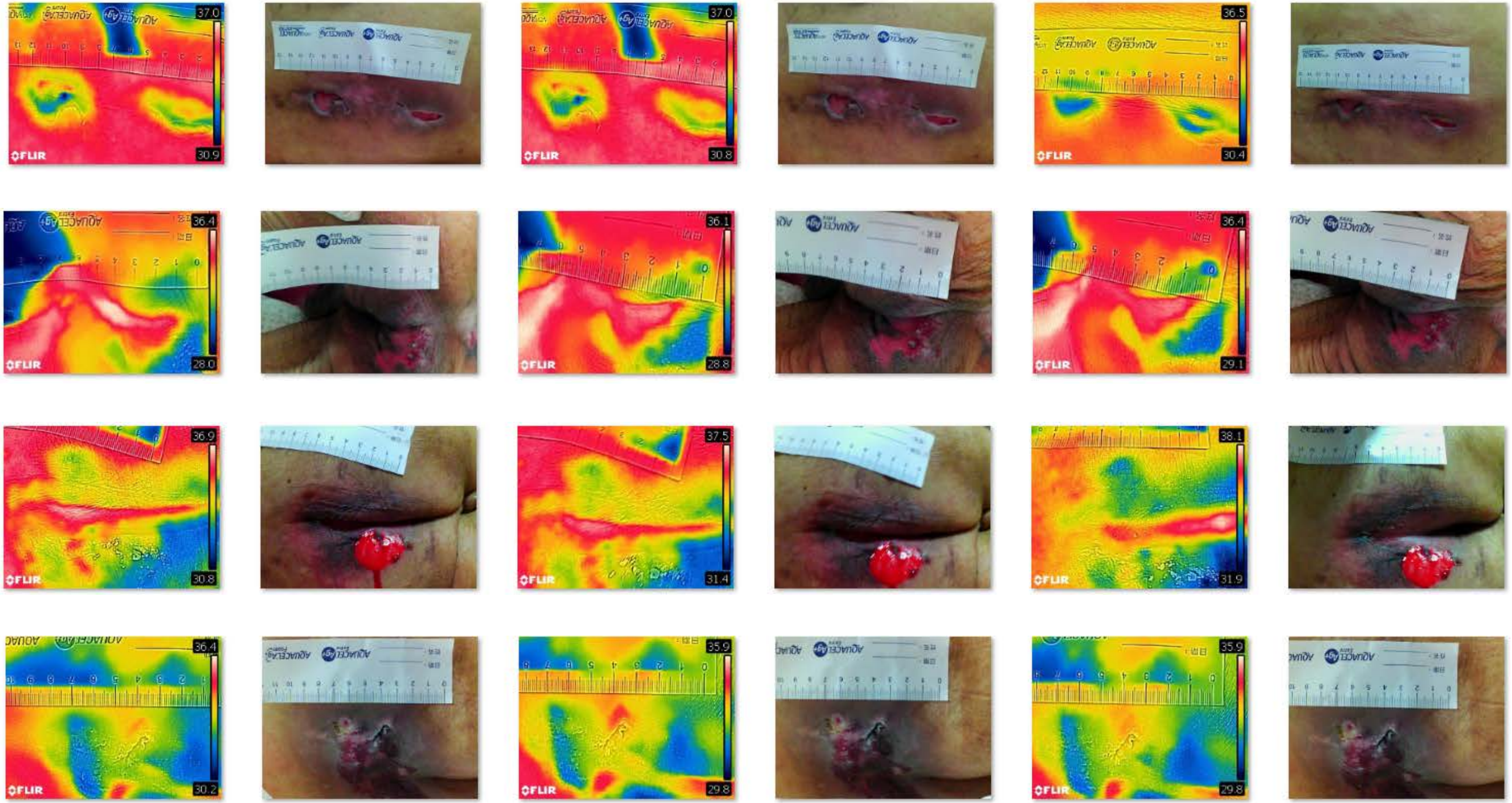
37.0
30.9
FLIR
37.0
30.8
FLIR
36.5
30.4
FLIR
36.4
28.0
FLIR
36.1
28.8
FLIR
36.4
29.1
FLIR
36.9
30.8
FLIR
37.5
31.4
FLIR
38.1
31.9
FLIR
36.4
30.2
FLIR
35.9
29.8
FLIR
35.9
29.8
FLIR

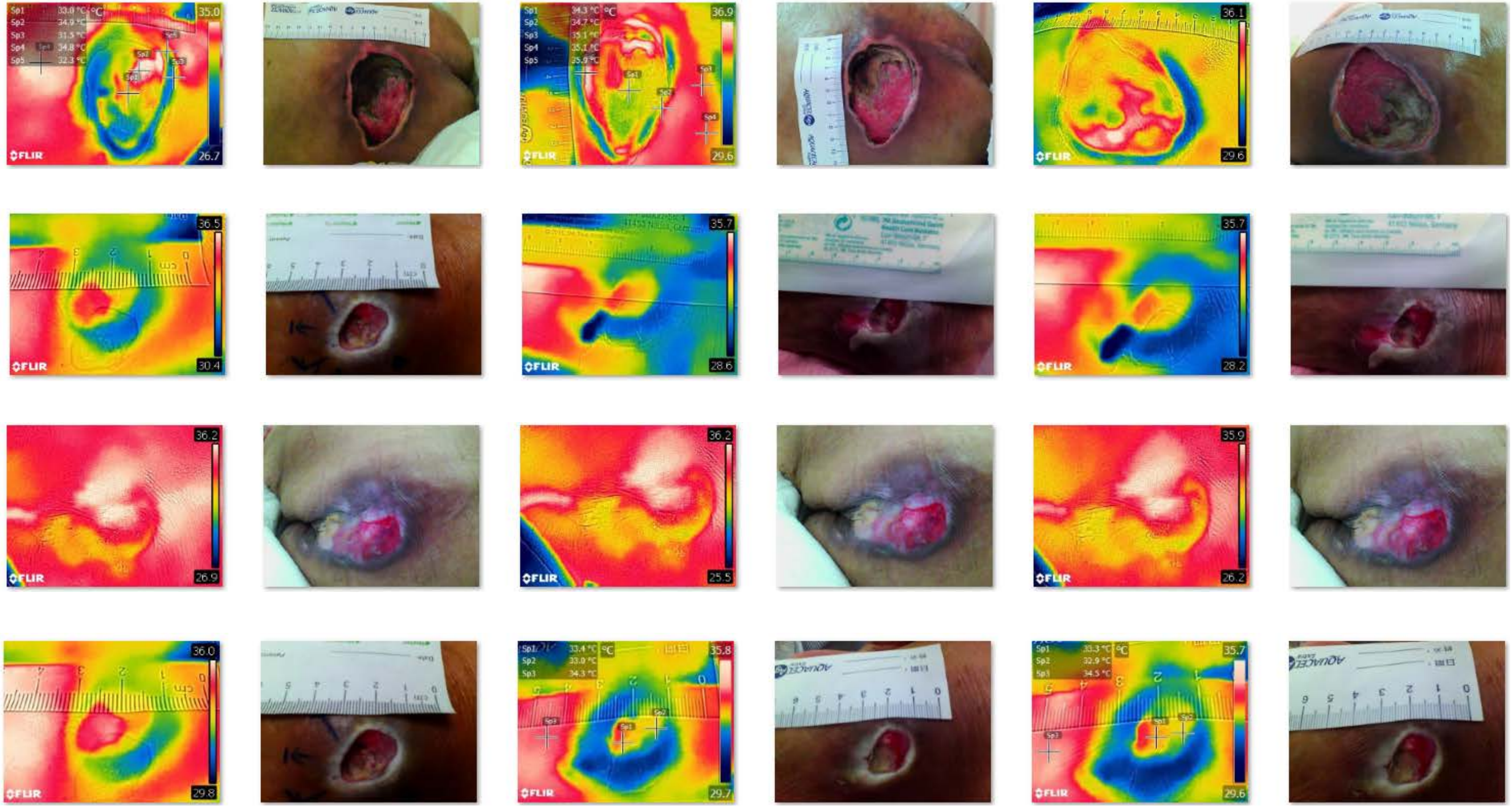
FLIR
35.0
26.7
36.9
29.6
36.1
29.6
36.5
30.4
35.7
28.6
35.7
28.2
36.2
26.9
36.2
25.5
35.9
26.2
36.0
29.8
35.8
29.7
35.7
29.6

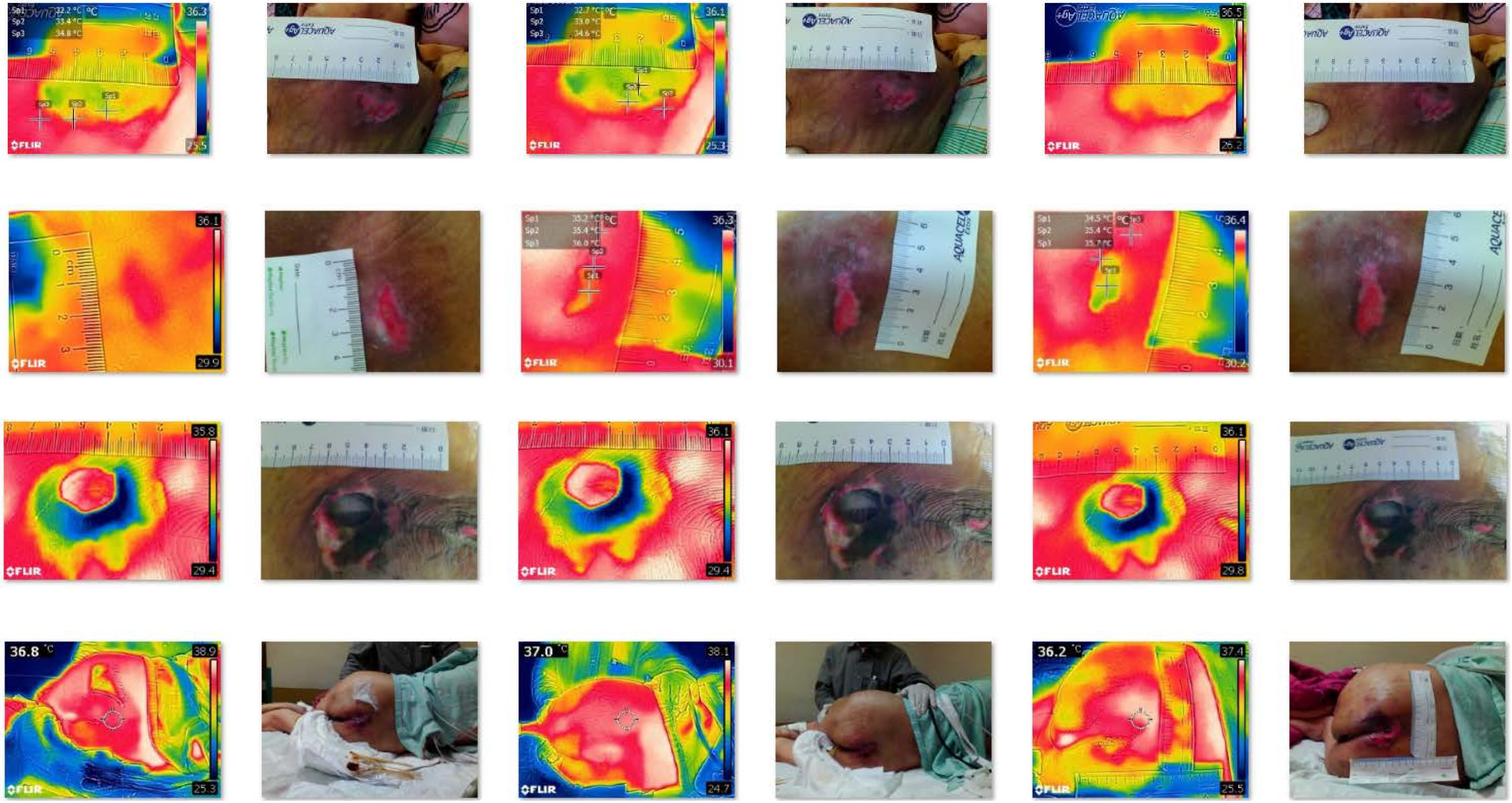
FLIR
36.3
25.5
36.1
25.3
36.5
26.2
36.1
29.9
36.3
30.1
36.4
30.2
35.8
29.4
36.1
29.4
36.1
29.8
36.8 °C
38.9
25.3
37.0 °C
38.1
24.7
36.2 °C
37.4
25.5
AQUACEL

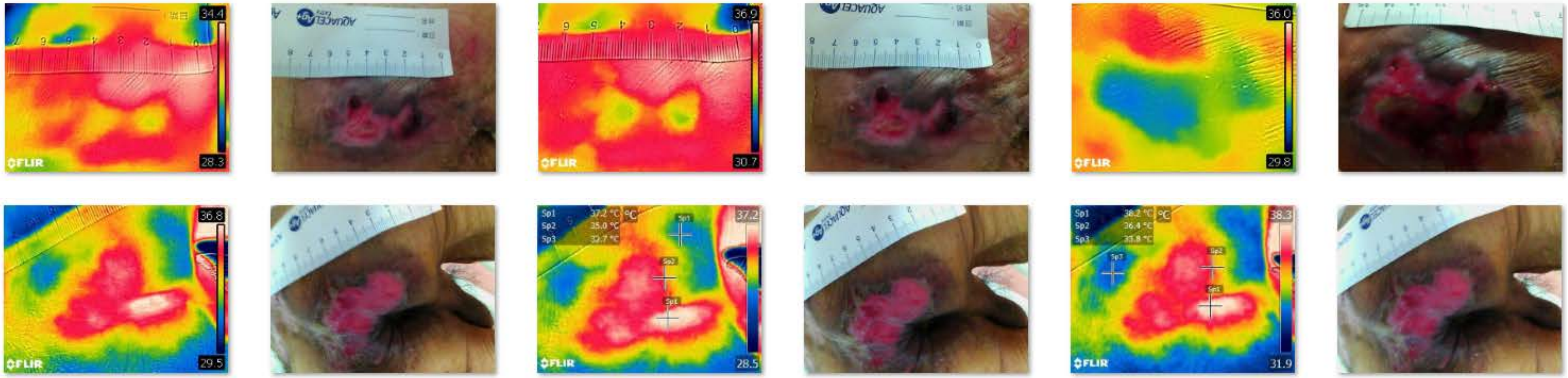
34.4
28.3
FLIR
AQUACEL Ag+
36.9
30.7
FLIR
AQUACEL
36.0
29.8
FLIR
36.8
29.5
FLIR
Sp1
Sp2
Sp3
37.2
28.5
FLIR
Sp1
Sp2
Sp3
38.3
31.9
FLIR

Figure S2: Case demonstrations of infected wound

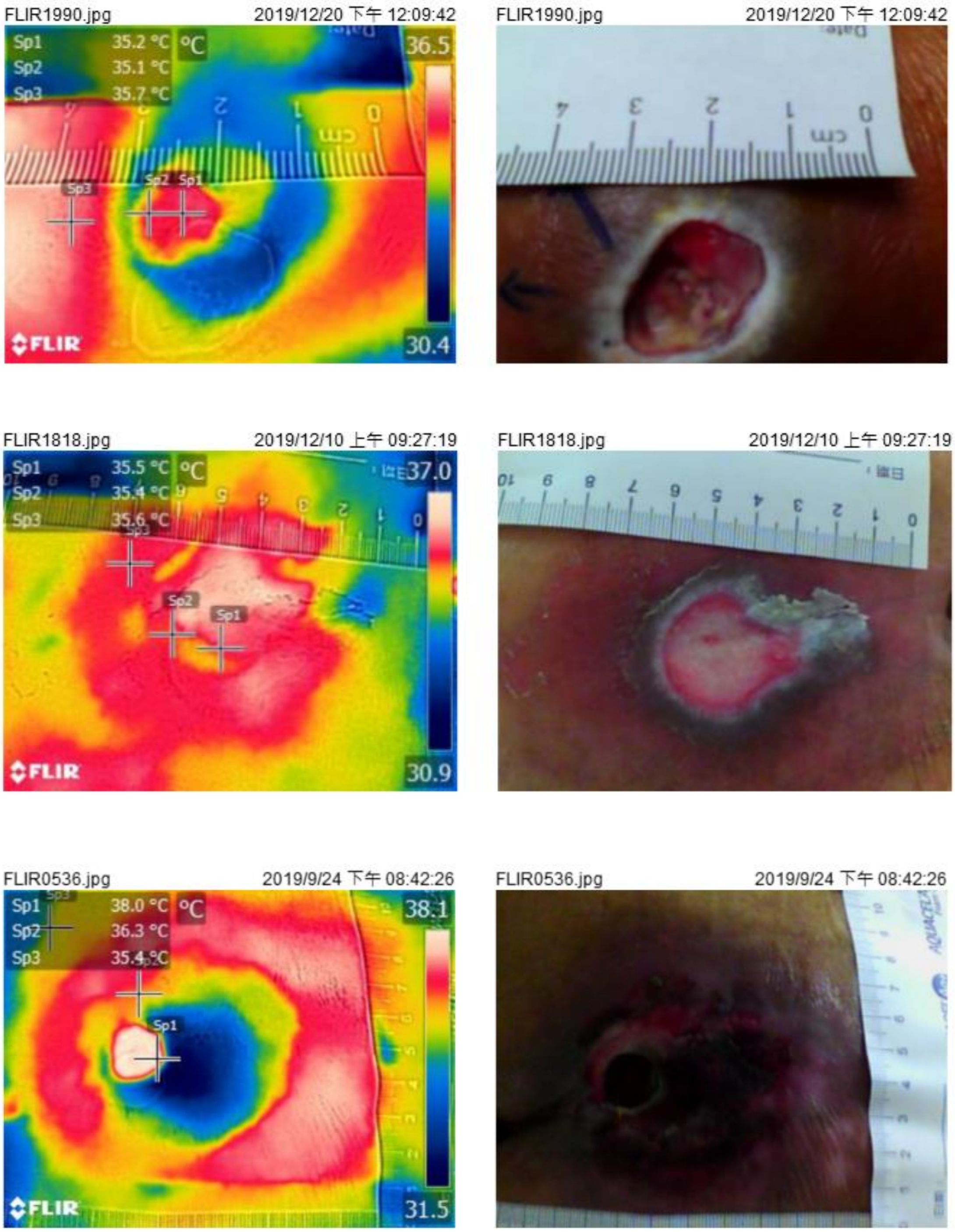

Supplement: Supplementary file 1 [file jcm-10-02883-s001.zip › jcm-1265732-supplementary.pdf]
